# Coal: in a burning world, the dark side of energy still rules

**DOI:** 10.7189/jogh.15.03007

**Published:** 2025-02-14

**Authors:** Helotonio Carvalho

**Affiliations:** 1 Department of Biophysics and Radiobiology, Biological Sciences Center, Federal University of Pernambuco, Recife, Pernambuco, Brazil; 2 Department of Immunology, Aggeu Magalhães Institute (IAM), Oswaldo Cruz Foundation (FIOCRUZ), Recife, Pernambuco, Brazil

## Abstract

The world registered record temperatures in the last years, with 2024 being the hottest year ever recorded and the first one to surpass the 1.5ºC limit defined by the Paris Agreement. Coal was key to the Industrial Revolution and along with petroleum, was essential to world development. However, coal is the most pollutant of fossil fuels, generating more CO_2_ and particulate material. Coal-derived air pollution is associated with several diseases including respiratory diseases such as chronic obstructive pulmonary disease and lower respiratory infections, cerebrovascular disease, ischaemic heart disease and lung cancer. Air pollution caused by coal and other fossil fuels causes millions of deaths a year. Despite its negative impacts on human health and climate change, coal has been extensively used for electricity generation in the last four decades and is still responsible for more than 35% of all the electricity produced in the world, with countries like Australia, Indonesia, Poland, India and China showing a much higher coal dependency from 45% to 75% in 2023. However, countries like UK, Denmark, Portugal and Spain heavily reduced coal use showing that a transition away from coal is possible and could be used by other nations.

The year 2023 saw record temperatures all over the world, with median temperatures close to the Paris Agreement limit of 1.5°C above the pre-industrial limits [1]. Higher temperatures have direct adverse health effects, contributing to an increase in deaths caused by heat stress as high as 35% for each 1°C of increased temperature [2]. Another consequence is the higher frequency and severity of droughts. In October 2023, severe droughts caused by higher temperatures affected the Amazon, contributing to a huge increase in the number of wildfires in the region, resulting in Manaus, the capital of Amazonas State, registering one of the worst air qualities in the world. While this poor air quality has severe effects on human health, drought consequences extend beyond, impairing the heavily river-dependent transport between Amazon cities [3]. Globally, climate change has been associated to a 15.8% increase in the area burned by wildfires between 2003–19 [4]. The year 2024 ended up with even higher median temperatures and was the first year to surpass the 1.5°C limit, reaching 1.6°C above the pre-industrial period [5]. Climate change heavily contributes to these extreme events and the increases in their frequency and intensity; simultaneously, the impacts of burning fossil fuels to climate change and to human health have been described for decades.

Coal was essential for the Industrial Revolution in the 18th century and reigned almost absolute for energy generation until petroleum began being used in the 19th century. Even though both are fossil fuels, coal has a much higher environmental and health impact, generating more CO_2_ and air pollution, mainly particulate material (PM_2.5_ and PM_10_), than any other fossil fuel [6]. According to the International Energy Agency, global energy-related CO_2_ emissions reached a new record high over 37.4 Gt in 2023 [7]. Air pollution derived from burning fossil fuels causes adverse health effects, including several diseases like cerebrovascular disease, ischaemic heart disease, chronic obstructive pulmonary disease, lower respiratory infections, and lung cancer [8]. Despite efforts to curb air pollution, more than seven million people die each year from diseases associated with air pollution [9]. The WHO estimates that, in 2019, 99% of the world population was living in areas which did not meet its newest air quality guidelines [10,11]. According to Cohen and colleagues [8], 17.1% of all deaths caused by PM_2.5_ were due to cardiovascular disease, 14.2% due to cerebrovascular disease, 16.5% due to lung cancer, 24.7% due to lower respiratory infections, and 27.1% due to chronic obstructive pulmonary disease. If we consider only air pollution generated by coal use for power generation, it is estimated to cause 52 000 deaths annually in the USA, 670 000 in China, and between 80 000 and 115 000 in India [12].

Health impacts of coal combustion occur due to gaseous components such as CO_2_, CO, NO_x_, and SO_x_, as well as particulate matter, which is a complex mixture formed by elemental carbon, metals, organic compounds and inorganic anions [13]. Coal is usually rich in sulphur, generating also SO_2_ and other sulphur compounds during combustion. Exposure to high levels of SO_2_ can cause acute respiratory symptoms like coughing, suffocation, and reduction in lung function. Systemically, SO_2_ reaction with mucous lining in the respiratory tract generates derivatives of mucin and surface glycans with a linked SO_3_^−^, which, through the blood stream, reach other organs and are associated to lung and colon cancer. SO_2_ can also promote bronchitis and cystic fibrosis due to the formation of sulphated glycans [14].

NO_2_ is associated with reduced pulmonary function and asthma. *In vivo*, NO_2_ forms peroxynitrite, a reactive nitrogen compound which causes oxidative stress inducing DNA mutations and protein alterations, which can ultimately result in cancer. Besides these direct effects, SO_2_ also contributes to the formation of particulate matter, the air pollutant that mostly impacts human health. The main mechanisms that contribute to adverse health effects of air pollution are the production of reactive oxygen species (ROS) and inflammation. PM_2.5_ is especially linked to systemic adverse responses, since it penetrates deeper in the respiratory tract compared to PM_10_, reaching the lung alveoli and the bloodstream. Besides the strong correlation between PM_2.5_ exposure and lung cancer, epidemiological data suggest an association with other types of cancer such as oesophagus, stomach, colon, liver, pancreas cancer, bladder, kidney, breasts, leukaemia, lymphoma, and the brain [13]. NO_2_ is also associated with breast and colorectal cancer, as well as lymphoma. However, associations between air pollution and cancer types other than lungs have not always been corroborated across different studies.

ROS production and inflammation also have a clear contribution in cardiovascular diseases, inducing lipid peroxidation and oxidative alterations in DNA, as well as impairment of endothelial cell function [15]. Particulate matter mediates superoxide production which reacts with NO producing peroxynitrite. Besides its oxidative potential, peroxynitrite production reduces NO availability, impairing its capacity to control vasodilation and blood pressure, inhibiting platelet aggregation and regulating inflammatory cells with impacts for cardiovascular diseases such as atherosclerosis and myocardial infarction.

One of the main uses of coal is electricity generation. According to the Energy Institute, during most of the last four decades, coal burning produced 35% to 40% of the electricity in the world [16]. The purpose of this viewpoint was to show the difference between countries that gave priority to reducing coal dependency and those which did not. Some countries have largely reduced their coal dependence over the years (**Figure 1**, Panel A). According to the Energy Institute’s analysis, coal burning generated 91.0% of electricity in Denmark in 1991, compared to only 7.9% in 2023. In the same period, the share of electricity generated by coal in Greece went down from 72.0% to 9.6%. In 1985, 57.0% of the electricity generation in the US came from coal, compared to 15.9% in 2023. One of the best examples for other coal-dependent nations is UK: between 1985 and 2023, the share of electricity generation in the country went down from 59.0% to 1.4%, and the last coal power plant was turned off in 30 September 2024 [17]. Almost as impressive is the case of Spain, for which the dependency on coal went from 43.0% to 1.4% in the same period, or Portugal, which virtually abolished the use of coal electricity generation since 2022. Hungary, Ireland, or Slovakia also managed to achieve substantial decreases in their coal dependency over the last decades [16].

**Figure 1:**
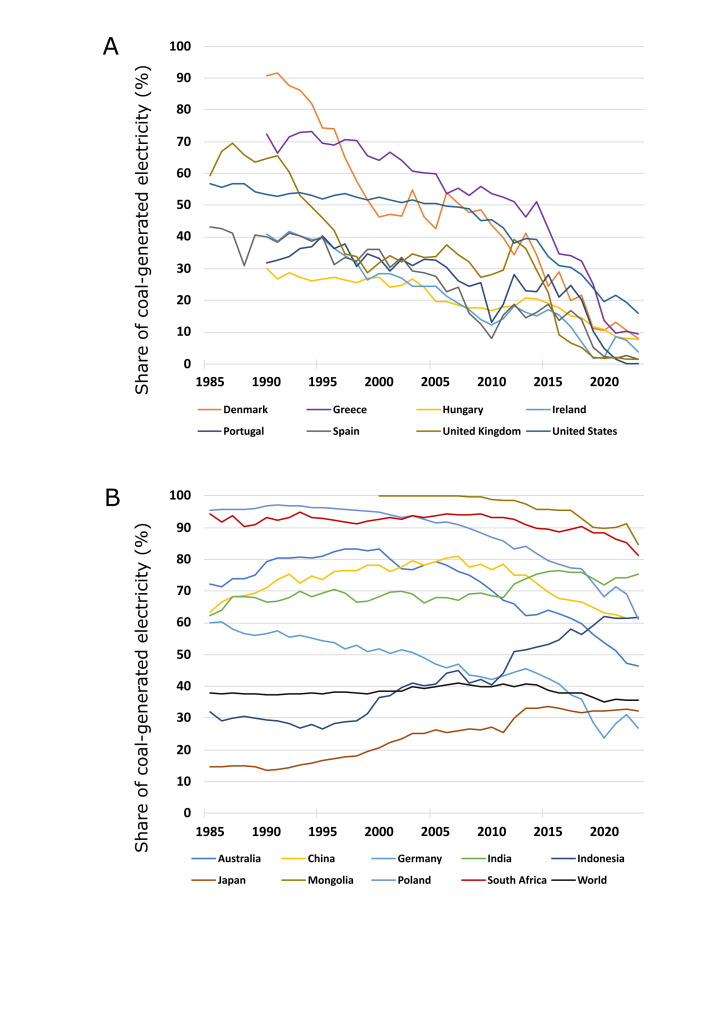
Coal use for electricity generation in selected countries in 1985–2023. Data obtained from the Energy Institute, with major processing by Our World in Data [15]. **Panel A.** High achievers. **Panel B.** High users.

In other countries, however, coal dependency is still high, according to the Energy Institute (**Figure 1**, Panel B) [16]. This is the case of Australia, which, despite the decrease in the share of electricity produced from coal, still relied on it for 46.0% of the electricity produced in 2023, or South Africa, which used coal for 81.0% of its power generation in the same year. Germany has nearly halved the share of electricity produced by coal burning, but it is still quite dependent on it (27.0%). In fact, Germany needed to increase electricity production from coal again in 2021–22 due to the Ukraine war, which limited its natural gas delivered from Russia. Almost all electricity in Poland (96.0%) was produced from coal in 1985; despite the decrease in the last decades, this proportion remains very high (61.0%).

In China, despite heavy investments in renewable energy, its increasing energy demand makes it contribute more to coal energy capacity to the world, with two new coal power plants opened per week in 2022 [18]. Thus, despite the drop in the share of electricity generated by coal since the peak of 81.0% in 2007, the decrease has been too slow, with the proportion remaining at 61.0% in 2023. India is another country with more planned coal power plants [19], which is why its share of electricity generated by coal has been increasing over the last decades, staying above 70% since 2012, despite an apparent stabilization in recent years. Indonesia has also been increasing its use of coal for electricity generation over the years, with an apparent stabilization at 61% only after 2020. In Japan, coal burning generated 25% of the electricity in 2011, the year of the Fukushima nuclear reactor meltdown. After that, all nuclear reactors were turned off [20] and, as a result, coal use started increasing again, reaching 32% in 2023. During most of the last two decades, Mongolia relied almost entirely on coal, and this fuel still responded for 85% of power generation in 2023. Other countries such as Bosnia and Herzegovina, Serbia, and Kazakhstan still used coal for at least 60% of their electricity generation in 2023 [16].

Most of coal dependency nowadays comes from power plants planned or put into operation decades ago, which has been driven either by coal abundancy or its low price. Even with the increasing investments in renewable energy sources in the last two decades, which induced a huge drop in solar and wind energy prices, the share of coal used for electricity generation in the world has remained almost unchanged in the last four decades at a little less than 40%, mainly due to new large added coal power capacities in China, India, and Indonesia. While renewable energy sources have been playing an important role in reducing coal dependency in some countries, this has not yet been able to offset, on a global scale, the increase in coal-powered energy production in these three countries. It should also be noted that, for some of the countries which drastically reduced coal dependency, the change does not always represent only an increase in energy production from renewables. This is the case for UK, where the share of electricity production from gas in the last 30 years went from less than 2% to 40% [16]. Even though gas is much cleaner than coal, it still contributes to CO_2_ emissions as other fossil fuels. However, during the same period, UK has also increased in the same rate the share of electricity production from renewable energy sources.

Even with the advances in renewable energy sources, there are some challenges that impair a more massive transition from coal to renewables. Coal availability is one factor: countries with large coal reserves and established coal mining like Australia, China, India, Indonesia, and Poland [21] are more prone to use them, as they are a more readily available energy source. Political pressure is also associated to the use of coal especially in developing countries such as India, Indonesia, or Poland, mainly in terms of the pressure from labour unions which fear losing jobs and negative effects to the local or regional economies. Long-term contracts for coal power plants also play a role, since mandatory shutdowns would probably imply in high fines besides adding regulatory uncertainties which could reduce the interest of the private sector in investing in the country. Yet perhaps one of the main factors which impair a massive change from coal to cleaner energy sources are subsidies. According to the OECD, direct coal subsidies amounted to about USD 36 billion in 2022 in 82 selected countries, including OECD members [22]. However, since electricity is highly subsidised in many of the countries which heavily use coal, this adds up to USD 472 billion to this bill.

Coal power plants provide stable energy generation, while solar and wind are affected by sun and wind availability, and the price of battery systems for energy storage still need to fall substantially to allow for scalability. A change from coal to natural gas, as happened in the UK, is not always possible, since it depends either of domestic reserves or imports from other countries, demanding the construction of long gas pipelines. Besides this, gas prices are usually higher than coal, limiting its adoption. Another factor which makes the change usually difficult is the necessary and expensive retrofit from coal to gas power plants.

High coal users could follow the example of some of the high achievers like the UK, which replaced coal with the less pollutant gas and also managed to heavily improve the use of renewable energy sources such as solar and wind, which accounted for 4.6% and 28.1%, respectively, of electricity production in 2023. With even higher shares of renewables planned for the following years, gas will be probably used as a backup system for periods when renewables have lower electricity production. Thus, for some high users at least, the use of gas can be an intermediate way out of fossil fuels, until they manage to build a larger infrastructure for renewables and can eventually opt for gas only when strictly necessary. Denmark has managed to achieve such a transition and, in 2023, used gas for only 2.5% of its electricity generation, down from 24.5% in 2000, while increasing the share of wind power from 11.8% to 57.7% in the same period [23]. These countries show that the transition away from coal is possible. Additionally, the cost of wind and solar power is already lower than coal [24], which should favour them for new projects, especially in low- and middle-income countries. Even with these lower costs, it may be necessary to utilise subsidies for renewables to get traction in some countries until the market is large enough to work properly on its own. A good example is the incentive for rooftop solar energy, which has been used in many countries and help reduce energy dependence from power plants. Another important policy would be to stop coal subsidies which have been used even in countries with a largely clean energy matrix like Brazil. According to the International Monetary Fund, subsidies for all fossil fuels were about USD 7.0 trillion in 2022, representing 7.1% of the global gross domestic product [25]. These subsidies do not make any sense while the world is trying to fight climate change, and these resources should be directed to renewable energy sources.

Unfortunately, the latest United Nations Climate Change Conferences (COP) have not contributed much to changing this situation. For example, COP28 has not come to an agreement on coal ban and just mentioned a ‘transition away’ from fossil fuels. The last one, COP29, agreed on increasing the funds to help energy transition in low- and middle-income countries from USD 100 billion to USD 300 billion a year until 2035, a value that was highly criticised for being insufficient. A list of countries, mostly developed ones (including UK, Canada, France, Germany, and Australia), finally agreed on not opening new coal power plants if they are not equipped with carbon capture technology, but this will have limited impact since most projects for new coal power plants take place in China and India, which did not sign the agreement.

The world needs more ambitious goals to limit climate change; a coal ban is a necessary and urgent step in this direction, a measure that will make a crucial contribution to reducing air pollution and help save millions of lives all over the world.

## References

[1] Copernicus. 2023 is the hottest year on record, with global temperatures close to the 1.5°C limit. 9 January 2024. Available: https://climate.copernicus.eu/copernicus-2023-hottest-year-record. Accessed: 25 June 2024.

[2] Faurie C Varghese BM Liu J Bi P Association between high temperature and heatwaves with heat-related illnesses: A systematic review and meta-analysis. Sci Total Environ. 2022;852:158332. 10.1016/j.scitotenv.2022.158332 36041616

[3] Watts J. Drought turns Amazonian capital into climate dystopia. Guardian. 18 October 2023. Available: https://www.theguardian.com/environment/2023/oct/18/drought-amazon-capital-climate-manaus-forest-fires-air-quality-rivers. Accessed: 25 June 2024.

[4] Burton C Lampe S Kelley DI Thiery W Hantson S Christidis N Global burned area increasingly explained by climate change. Nat Clim Chang. 2024;14:1186–92. 10.1038/s41558-024-02140-w

[5] Poynting M, Rivault E, Dale B. 2024 first year to pass 1.5C global warming limit. 10 January 2025. Available: https://www.bbc.com/news/articles/cd7575x8yq5o. Accessed: 24 January 2025.

[6] US Energy Information Administration. Carbon Dioxide Emissions Coefficients. 18 September 2024. Available: https://www.eia.gov/environment/emissions/co2_vol_mass.php. Accessed: 29 June 2024.

[7] International Energy Agency. CO_2_ Emissions in 2023. March 2024. Available: https://www.iea.org/reports/co2-emissions-in-2023. Accessed: 29 June 2024.

[8] Cohen AJ Brauer M Burnett R Anderson HR Frostad J Estep K Estimates and 25-year trends of the global burden of disease attributable to ambient air pollution: an analysis of data from the Global Burden of Diseases Study 2015. Lancet. 2017;389:1907–18. 10.1016/S0140-6736(17)30505-6 28408086 PMC5439030

[9] World Health OrganizationAir Pollution. 2025. Available: https://www.who.int/health-topics/air-pollution#tab=tab_1. Accessed: 25 June 2024.

[10] World Health Organization. Ambient (outdoor) air pollution. 2025. Available: https://www.who.int/news-room/fact-sheets/detail/ambient-(outdoor)-air-quality-and-health. Accessed: 22 June 2024.

[11] Carvalho H New WHO global air quality guidelines: more pressure on nations to reduce air pollution levels. Lancet Planet Health. 2021;5:e760–1. 10.1016/S2542-5196(21)00287-4 34774116

[12] Hendryx M Zullig KJ Luo J Impacts of Coal Use on Health. Annu Rev Public Health. 2020;41:397–415. 10.1146/annurev-publhealth-040119-094104 31913772

[13] Turner MC Andersen ZJ Baccarelli A Diver WR Gapstur SM Pope CA III Outdoor air pollution and cancer: An overview of the current evidence and public health recommendations. CA Cancer J Clin. 2020;70:460–79. 10.3322/caac.21632 32964460 PMC7904962

[14] Munawer ME Human health and environmental impacts of coal combustion and post-combustion wastes. J Sustain Min. 2018;17:87–96. 10.1016/j.jsm.2017.12.007

[15] Miller MR The cardiovascular effects of air pollution: Prevention and reversal by pharmacological agents. Pharmacol Ther. 2022;232:107996. 10.1016/j.pharmthera.2021.107996 34571110 PMC8941724

[16] Richie H, Rosado P. Fossil fuels. Our World in Data. January 2024. Available: https://ourworldindata.org/fossil-fuels. Accessed: 14 November 2024.

[17] Ambrose J. End of an era as Britain’s last coal-fired power plant shuts down. Guardian. 30 September 2024. Available: https://www.theguardian.com/business/2024/sep/30/end-of-an-era-as-britains-last-coal-fired-power-plant-shuts-down. Accessed: 18 October 2024.

[18] Myllyvirta L, Yu A, Champenois F, Zhang X. China permits two new coal power plants per week in 2022. Centre for Research on Energy and Clean Air. 27 February 2023. Available: https://energyandcleanair.org/publication/china-permits-two-new-coal-power-plants-per-week-in-2022/. Accessed: 23 June 2024.

[19] Behl M. After China, India has most proposed coal-powered plants: Report. Times of India. 26 April 2022. Available: https://timesofindia.indiatimes.com/city/nagpur/after-china-india-has-most-proposed-coal-powered-plants-report/articleshow/91083432.cms. Accessed: 24 January 2025.

[20] Jonathan S, Russo J, Zaretskaya VUS. Nuclear reactor restarts in Japan have reduced LNG imports for electricity generation. Energy Information Administration. 2024. Available: https://www.eia.gov/todayinenergy/detail.php?id=61386. Accessed: 23 June 2024.

[21] US Energy Information Administration. Coal reserves 2023. Available: https://www.eia.gov/international/rankings/world?pa=264&u=0&f=A&v=none&y=01%2F01%2F2023&ev=fals. Accessed: 9 February 2025.

[22] Organisation for Economic Co-operation and Development. Fossil fuel support. Available: https://www.oecd.org/en/topics/fossil-fuel-support.html. Accessed: 5 November 2024.

[23] Ember, Energy Institute – Statistical Review of World Energy - with major processing by Our World in Data. 2024. Available: https://ourworldindata.org/grapher/share-electricity-source-facet. Accessed: 26 November 2024.

[24] Roser M. Why did renewables become so cheap so fast? Our World in Data. 1 December 2020. Available: https://ourworldindata.org/cheap-renewables-growth. Accessed: 26 November 2024.

[25] International Monetary Fund. Fossil Fuel Subsidies. Available: https://www.imf.org/en/Topics/climate-change/energy-subsidies. Accessed: 29 November 2024.

